# Small-polaron transport in perovskite nickelates

**DOI:** 10.1038/s41598-023-39821-z

**Published:** 2023-08-01

**Authors:** M. Tyunina, M. Savinov, O. Pacherova, A. Dejneka

**Affiliations:** 1 Institute of Physics of the Czech Academy of Sciences, Na Slovance 2, 18220 Prague, Czech Republic; 2 Microelectronics Research Unit, Faculty of Information Technology and Electrical Engineering, University of Oulu, P. O. Box 4500, Fl-90014 Oulu, Finland

**Keywords:** Condensed-matter physics, Materials for devices

## Abstract

Knowledge of the explicit mechanisms of charge transport is preeminent for a fundamental understanding of the metal-to-insulator transition in *AB*O_3_-type perovskite rare-earth nickelates and for potential applications of these technologically promising materials. Here we suggest that owing to intrinsic Jahn–Teller-driven carrier localization, small-polaron transport is innate in nickelates. We demonstrate experimental evidence for such transport by investigating AC conductivity over a broad range of temperatures and frequencies in epitaxial SmNiO_3_ films. We reveal the hopping mechanism of conductivity, Holstein-type activation energy for hopping, nonclassical relaxation behavior, and nonclassical consistency between activation and relaxation. By analyzing these observations, we validate small-polaron transport. We anticipate that our findings can lead to precise tailoring of the DC and AC conductivity in nickelates as requested for fruitful employment of these materials. We also believe that further investigations of self-trapped small polarons are essential for a comprehensive understanding of nickelates.

## Introduction

*AB*O_3_-type perovskite-structure rare-earth nickelates (*RE*NiO_3_) are known for their metal-like electrical conductivity at high temperatures and orders-of-magnitude drop in conductivity (increase in resistivity) upon cooling^1–4^. This metal-to-insulator (MI) change in conductivity is associated with a complex phase transition (MIT), whose temperature *T*_*MIT*_ depends on the type of *RE* cation. Additionally, in thin nickelate films, the temperature *T*_*MIT*_ can vary with substrate-induced lattice strain and/or film thickness (down to a few unit cells)^5,6^. Being of fundamental interest and promising for emerging devices, MIT in nickelates has attracted significant research efforts. However, the explicit mechanisms of application-relevant charge transport in these materials are far from being completely understood.

For the high-temperature metal-like phase of *RE*NiO_3_, DC conductivity is normally rather small compared to that in typical metals, and the temperature dependence of the conductivity (resistivity) deviates from the classical behavior of metals. Although this deviation can be satisfactorily described in terms of Fermi liquid, non-Fermi liquid, or bad metal models^7–10^, its origin is still ambiguous.

Importantly, the reported magnitude of conductivity (for certain *RE* and temperature) is strongly scattered. These conductivity alterations are poorly explored despite their critical importance for potential applications. We note that conceivable devices are likely to employ changes (induced by applied electric field, irradiation with light, etc.) of the low-temperature conductivity. However, whereas investigations of the high-temperature behavior and *T*_*MIT*_ are vast, charge transport in the low-temperature insulator phase is largely uncharted. We also note that the coexistence of phases over a broad range of temperatures is characteristic of nickelates^11^, which makes the understanding of low-temperature transport essential for a better understanding of high-temperature conductivity as well. Here we scrutinize low-temperature insulator behavior.

For an insulator, in the presence of a band gap and the absence of band carriers (e.g. electrons in the conduction band), charge transport is possible by hopping between localized electron states^12–14^. Such states can be caused by structural defects or disorder, or chemical doping, have diverse energies, and possess statistical density-of-states (different from that in the conduction/valence band). More generally, localized and band states can coexist in disordered semiconductors. The related DC hopping conductivity increases with temperature as [*σ*_0_ ∝ exp(− *T*_*p*_/*T*)^*p*^], where the characteristic temperature *T*_*p*_ and the exponent [*p* ≤ 1] depend on the density of localized states near the Fermi level. An important distinctive feature of the hopping conductivity is its frequency dispersion, whereas the band conductivity is independent of frequency *f* <  < 10^14^ Hz^14^. The real part of AC hopping conductivity increases with frequency* f* as [*σ* ∝ *A*(*f*)^*s*^], where the relaxation exponent [*s* ≤ 1] decreases linearly with temperature [*s* ∝ -*T*] for classical barrier hopping^14^.

In contrast to carrier localization induced by defects or disorder, the self-trapping of carriers can naturally occur in *AB*O_3_-type perovskite-structure metal oxides^15–22^. The self-trapping phenomenon and the formation of small polarons are enabled by Jahn–Teller effects, which are innate in *AB*O_3_ perovskites and do not require disorder. For *RE*NiO_3_ perovskites, the formation of small polarons (SPs) was indicated in optical responses^7,23–25^. However, the contribution of SPs to charge transport in nickelates remains elusive^26^. Here we deliver experimental evidence for SP conductivity.

SP transport is realized by hopping, which is determined by phonons and electron–phonon coupling and, strictly, differs from the abovementioned conventional disorder-induced hopping^27–30^. SP DC conductivity behaves as [*σ*_0_ ∝ *T*^*–*1^⋅exp(− *E*_*A*_/*T*)], where the hopping activation energy *E*_*A*_ may vary with temperature, phonon frequency, and electron–phonon coupling in *AB*O_3_ perovskites^31^. Concurrently, frequency-dependent SP AC conductivity may not exhibit classical relaxation due to substantial electron–phonon coupling.

In this work, we experimentally demonstrate SP transport in epitaxial perovskite SmNiO_3_ (SNO) films. We selected such films because epitaxial growth of SNO on different substrates is well-established and the range of temperatures for the insulator phase is wide therein (*T*_*MIT*_ ≈ 400 K)^32–45^. To detect SP transport, the conductivity was studied in the broad range of temperatures from 8 to 500 K and frequencies from 1 to 10^6^ Hz.

We show the hopping mechanism of conductivity, prove the SP-specific temperature-dependent activation energy for hopping, detect a strain-modified phonon effect on the activation energy, and we reveal an unusual direct connection between the self-consistent relaxation parameters and the activation energy. Our observations corroborate SP transport.

## Materials and methods

Thin SNO films (thickness ~ 100 nm) were grown on epitaxially polished (001)LaAlO_3_ (LAO) and (001)(LaAlO_3_)_0.3_(Sr_2_AlTaO_6_)_0.7_ (LSAT) substrates purchased from MTI Corporation. The films were grown by pulsed laser deposition using a KrF excimer laser (energy density ~2 J/cm^2^)^46,47^. A substrate temperature of 973 K was kept during deposition and lowered at a rate of 5 K·min^–1^ during post-deposition cooling. The oxygen pressure was 20 Pa during deposition and post-deposition cooling. The crystal structure of the films was studied by high-resolution x-ray diffraction on a D8 DISCOVER diffractometer (Bruker corporation) using Cu Kα radiation (wavelength 1.5406 Å). The lattice parameters were estimated from the positions of the diffraction maxima using the substrates as a reference. The diffraction data were fitted using LEPTOS software.

In terms of pseudocubic perovskite cells, the films are epitaxial, with the (001) planes and the [100] directions parallel to those of the substrates, the in-plane lattice parameters *a*_*F*_ close to those of the substrates, and the out-of-plane lattice parameters *c*_*F*_ ~ 3.945 Å on LAO and ~ 3.911 Å on LSAT (Supplementary Figs. S1–S3). Compared to the room-temperature monoclinic phase of bulk SNO^4^, the films experience anisotropic lattice strain, which can be roughly characterized by the relative change in perovskite unit-cell volume: [*δV* = (*V*_*F*_/*V*_*BULK*_–1)]. Here *V*_*BULK*_ is the volume of perovskite subcell in bulk SNO and the volume *V*_*F*_ is [*c*_*F*_ × (*a*_*F*_)^2^] in the films. The relative expansion *δV* is ~ 3.6% in SNO/LAO and ~ 6.9% in SNO/LSAT, which qualitatively agrees with the complex substrate-induced changes in the lengths and angles of the Ni–O–Ni bonds in epitaxial nickelate films^48^.

For electrical characterization, Au stripes of 1 mm in widths and with 2 mm separation between them were formed by vacuum pulsed laser deposition using a shadow mask, and the samples of 3 mm in width were prepared (Supplementary Fig. S4). The in-plane AC conductivity was accurately assessed by measuring the small-signal impedance, where the contribution of substrates was insignificant owing to negligible dielectric losses in LNO and LSAT^49^.

The impedance was measured using a NOVOCONTROL Alpha-AN High Performance Frequency Analyzer. As a probing small signal, we used the sinusoidal ac voltage with the amplitude of 1–10 mV and the frequency *f* = (1–10^6^) Hz. The control of temperature was realized using a JANIS ST-100 He flow cryostat equipped with a LakeShore 335 temperature controller. The temperature was swept between 8 and 500 K at a rate of 5 K/min. The measurements were performed during heating and cooling runs. The acquired data were analyzed using Origin software.

## Results and discussion

**Figure 1:**
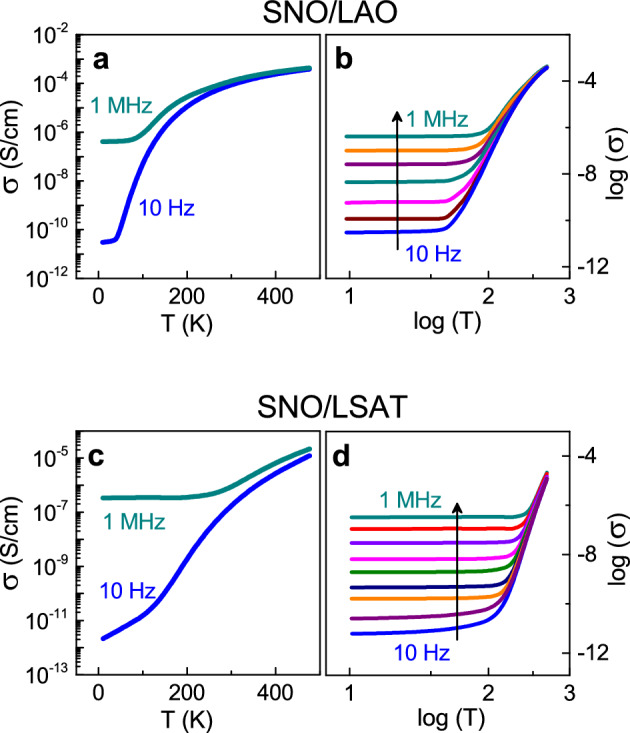
AC conductivity. (**a**,**c**) Semi-log and (**b**,**d**) log–log conductivity-temperature plots for different frequencies in the (**a**,**b**) SNO/LAO and (**c**,**d**) SNO/LSAT films. In (**b**,**d**), arrows show directions of frequency increase. The frequencies are 10, 10^2^, 10^3^, 1.7 × 10^4^, 5 × 10^5^, and 10^6^ Hz in (**b**) and 10, 4 × 10^2^, 10^3^, 8 × 10^3^, 3 × 10^4^, 2 × 10^5^, 7 × 10^5^, and 10^6^ Hz in (**d**).

The measured AC conductivity is found to increase with increasing temperature and/or frequency (Fig. 1). The lower-frequency conductivity *σ*(*T*) (see *f* = 10 Hz) exhibits a massive, several-orders-of-magnitude growth upon heating from 8 to 500 K (Fig. 1a,c). This behavior is consistent with the previously reported DC resistivity and indicates an insulator phase below 400 K^32–45^. Importantly, a strong frequency dispersion for the conductivity is evident below room temperature (Fig. 1b,d). At *T* = 10 K, the ratio of conductivity at *f* = 1 MHz to that at *f* = 10 Hz is immense: ~ 10^4^ in SNO/LAO and ~ 10^5^ in SNO/LSAT. The observed temperature- and frequency-dependent behavior *σ*(*T,f*) suggests a low-temperature insulator phase with hopping transport therein. The strong frequency dispersion and very small magnitude of conductivity imply negligible if any contribution of band transport^12–14^.

Because the low-frequency conductivity *σ*(*T*) is close to the DC behavior (Supplementary Fig. S5), we analyze its temperature dependence to distinguish SP transport. First, we assume conventional hopping conductivity (1),

1 $${\sigma }_{0}\left(T\right)={\alpha }_{0}exp{\left(-\frac{{T}_{p}}{T}\right)}^{p},$$

where the characteristic parameters *T*_*p*_ and *p* are specific for different regimes. The exponent *p* can take values of 1 (nearest-neighbor hopping, or NNH), 1/4 (variable-range hopping, or VRH, for the three-dimensional case), 1/3 (VRH for the two-dimensional case), 1/2 (Efros-Shklovski VRH, or ES). With increasing temperature, a sequence of transitions from ES to VRH and then to NNH can generally occur^13,14^.

The behavior of [ln(*σ*)] is analyzed as a function of [*T*^*–p*^] in the SNO/LAO and SNO/LSAT films (Supplementary Figs. S6 and S7). Although satisfactory fits to expression (1) with different values of *p* can be obtained therein, the fits are valid only in random narrow temperature ranges, and the theoretical ES-VRH-NNH (or VRH-NNH) sequence is absent. The revealed poor ES, VRH, or NNH fits and the absence of the ES-VRH-NNH sequence indicate that the hopping transport may not be defects-mediated in the SNO films. As shown below, the transport is by small polarons.

**Figure 2:**
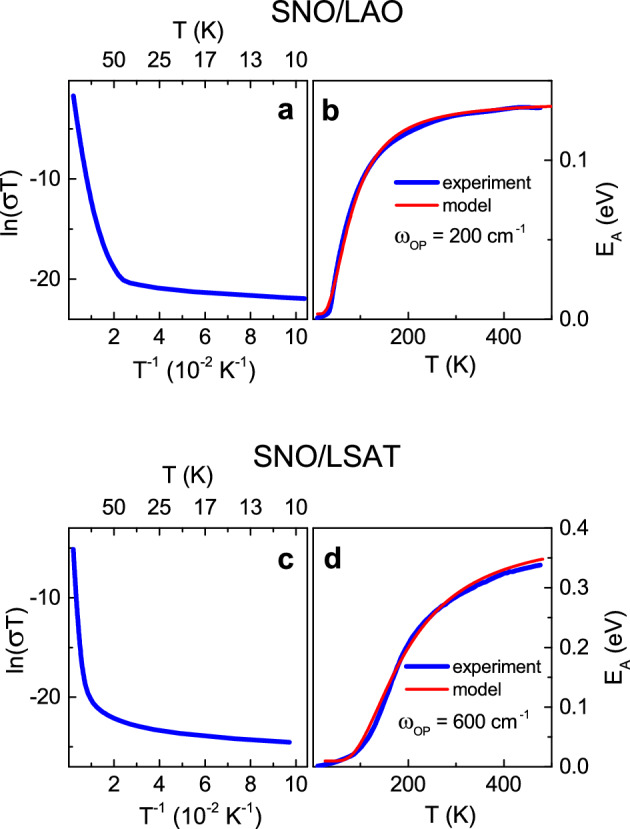
Conductivity (at 1 Hz). (**a**,**c**) Conductivity-temperature relationship [ln(*σT*) vs *T*^–1^] and (**b**,**d**) activation energy *E*_A_ as a function of temperature in the (**a**,**b**) SNO/LAO and (**c**,**d**) SNO/LSAT films. In (**b**,**d**), thick (blue) curves and thin (red) curves show the experimental and modeling results, correspondingly.

Next, we probe SP DC conductivity (2),

2 $${\sigma }_{0}={\alpha }_{0}{T}^{-1}exp\left(-\frac{{E}_{A}}{{k}_{B}T}\right),$$

where the hopping activation energy *E*_*A*_ may vary with temperature. We plot [ln(*σT*)] as a function of inverse temperature [*T*^–1^] in accordance with expression (2) (Fig. 2a,c) and extract the activation energy *E*_A_ by differentiation:

3 $${E}_{A}=-{k}_{B}\frac{d\left[ln\left(\sigma T\right)\right]}{d\left[{T}^{-1}\right]}.$$

The obtained experimental energy *E*_*A*_ grows with increasing temperature *T* (Figs. 2b,d). In terms of band conductivity, such an increase in *E*_*A*_ suggests bandgap widening at high temperatures, which is obviously not the case. The behavior of *E*_A_(*T*) is qualitatively consistent with the SP mobility. To scrutinize the dependence *E*_A_(*T*), we consider the theoretical (Holstein) SP mobility μ_SP_ (4)^29,30^:

4 $${\mu }_{SP}=\frac{e{a}^{2}{J}^{2}}{{k}_{B}T{\hslash }^{2}{\omega }_{OP}}{\left[\frac{\pi }{\gamma \mathrm{cosh}\left(\frac{{\hslash \omega }_{OP}}{4{k}_{B}T}\right)}\right]}^\frac{1}{2}exp\left[-2\gamma \mathrm{tanh}\left(\frac{{\hslash \omega }_{OP}}{4{k}_{B}T}\right)\right].$$

Here, *e* is the elementary charge, *a* is the lattice constant, *J* is the overlap integral,* k*_B_ is the Boltzmann constant, *ω*_OP_ is the frequency of the optical phonon, and *γ* is the electron–phonon coupling constant. Then the theoretical shape of *E*_A_(*T*) can be found by differentiating the plot of [ln(μ_SP_T)] versus [*T*^–1^]. The results of our simulations for the factor *ea*^2^*J*^2^ = 1, the coupling constant *γ* from 5 to 20, and the phonon frequency ω_OP_ from 30 to 1000 cm^–1^ are presented in (Supplementary Fig. S8). The modeled shape of the temperature dependence *E*_A_(*T*) is primarily determined by the magnitude of the phonon frequency ω_OP_ and weakly changes with the coupling constant *γ*. The model energy *E*_*A*_ is an increasing function of temperature and can tend to a steady value only for sufficiently small parameters ω_OP_ and *γ*.

The behavior of *E*_A_(*T*), extracted from the experimentally measured conductivity, can be well reproduced by model simulations using the coupling constant *γ* = 10 and different phonon frequencies ω_OP_: 200 cm^–1^ in SNO/LAO and 600 cm^–1^ in SNO/LSAT (Fig. 2b,d). We note that satisfactory agreement between the model and experiment can also be attained for other values of *γ* with slightly varied phonon frequencies. Importantly, for any employed *γ*, the frequency ω_*OP*_ in SNO/LSAT is found to be higher than that in SNO/LAO. This observation points to phonon hardening in SNO/LSAT compared to SNO/LAO. Previously, strain-induced phonon hardening was detected in other *AB*O_3_ perovskite films^50–55^. Here, the relative unit-cell expansion, which characterizes anisotropic lattice strain, is larger in SNO/LSAT. Correspondingly, a larger strain can lead to a higher frequency ω_*OP*_ in SNO/LSAT. It is worth noting that phonon hardening enhances the magnitude of *E*_A_ and makes *E*_A_(*T*) more sensitive to temperature variation over a wider temperature range (Supplementary Fig. S8 and Fig. 2b,d). The detected strain-modified phonon effect on the activation energy is specific for SP hopping. Our observations (Fig. 2) confirm SP transport in SNO.

Thus, the performed analysis of the temperature dependence of conductivity establishes the validity of relationships (2), (3) and suggests SP hopping. Moreover, it reveals the effect of strain on the energy *E*_A_(*T*), which agrees with strain-induced phonon hardening and corroborates SP transport. To further substantiate SP transport, we analyze frequency-dependent AC conductivity.

The hopping transport is manifested by a frequency dispersion of the real part of the AC conductivity *σ*, which is generally represented by (5)^13,14^:

5 $$\sigma ={\sigma }_{0}+A{f}^{s}.$$

Here *σ*_0_, *s* and *A* are the DC conductivity, relaxation exponent, and coefficient, correspondingly, all of which are temperature dependent. The coefficient *A* is proportional to DC conductivity and depends on relaxation mechanism^14^. For tunneling transport, the exponent *s* is close to unity *s* < 1, whereas for classical barrier hopping, the exponent *s* follows Pike’s law (6)^13,14^

6 $$s=1-\frac{6{k}_{B}T}{{E}_{H}} ,$$

where *E*_*H*_ is the barrier height, which can be approximated by the hopping activation energy.

**Figure 3:**
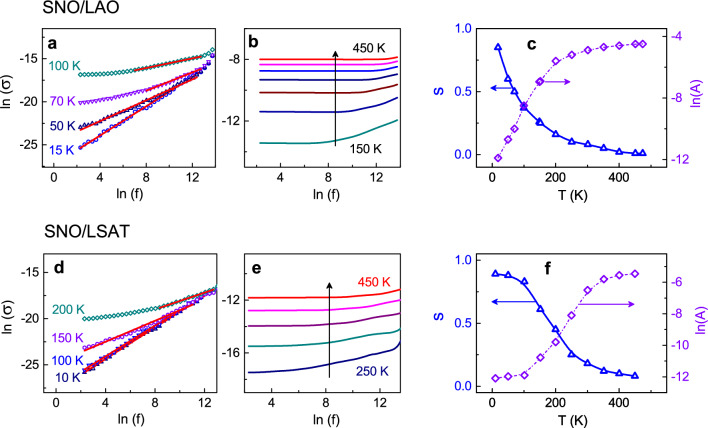
AC conductivity. (**a**,**b**,**d**,**e**) Log–log conductivity–frequency plots for different temperatures in the (**a**–**c**) SNO/LAO and (**d**–**f**) SNO/LSAT films. In (**a**,**d**), red lines show fits. In (**b**,**e**), arrows show directions of temperature increase with the step of 50 K. The temperatures are (**c**,**f**) The relaxation exponent *s* and coefficient ln(*A*) as a function of temperature in the (**c**) SNO/LAO and (**f**) SNO/LSAT films.

The experimental plots of [ln(*σ*) versus ln(*f*)], obtained for the SNO/LAO and SNO/LSAT films, are shown in (Fig. 3a,b,d,e). They contain linear fractions, in agreement with the hopping behavior (5). The parameters *s* and ln*A*, extracted from the linear fits [ln(*σ*) ∝ {ln*A* + *s*ln(*f*)}], are found to vary with temperature (Fig. 3c,f). The relaxation exponent is *s* < 1 and decreases with increasing temperature, which is qualitatively expected for hopping transport. At low temperatures, the exponent is close to 1 and indicates tunneling, which is also manifested by plateauing of conductivity (Fig. 1b,d). Importantly, the observed temperature-dependent behavior *s*(*T*) is not linear and, furthermore, dramatically differs from that calculated using Pike’s law (6) with the energy *E*_*H*_ equal to the experimentally determined activation energy *E*_*A*_ (Supplementary Fig. S9).

**Figure 4:**
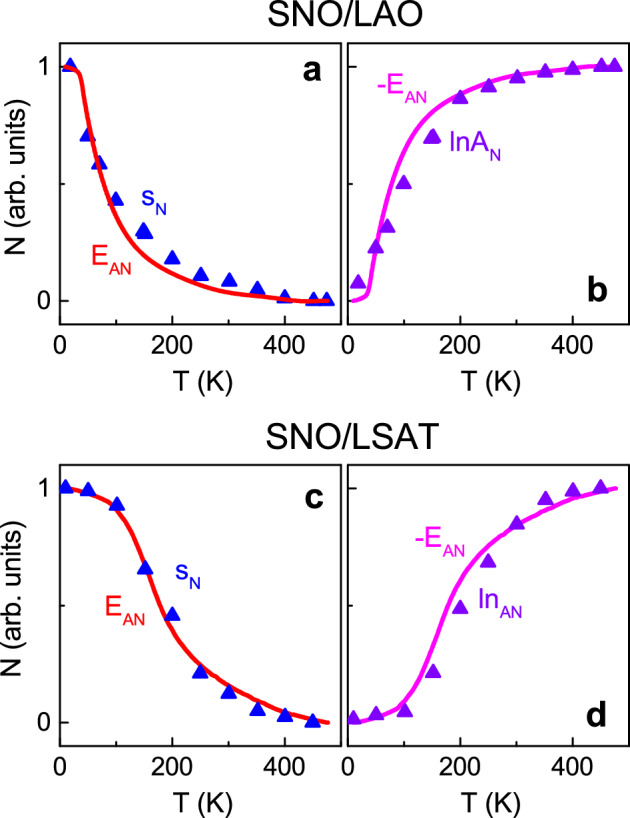
Relaxation parameters. The normalized parameters* N* as a function of temperature in the (**a**,**b**) SNO/LAO and (**c**,**d**) SNO/LSAT films. In (**a**,**c**), symbols and solid curves show the normalized relaxation exponent *s*_N_ and the negative normalized activation energy – *E*_AN_, correspondingly. In (**b**,**d**), symbols and solid curves show the normalized relaxation coefficient ln*A*_N_ and the normalized activation energy *E*_AN_, correspondingly.

To scrutinize the shapes of *s*(*T*) and ln*A*(*T*), the experimentally obtained parameters *s*, ln*A*, and *E*_*A*_ were normalized to vary between 0 and 1 for the studied temperatures (Fig. 4). The normalized relaxation exponent *s*_*N*_(*T*) is found to explicitly follow the negative normalized energy − *E*_*AN*_(*T*) in both SNO/LAO and SNO/LSAT (Fig. 4a,c). This observation signifies a direct correlation between the relaxation exponent and the SP activation energy *E*_*A*_:

7 $$s\left(T\right)=1-\kappa {E}_{A}\left(T\right),$$

where *κ* is the coefficient. The unveiled relationship (7) contrasts to Pike’s law (6). Concurrently, the normalized coefficient ln*A*_*N*_ practically coincides with the normalized energy *E*_*AN*_ (Fig. 4b,d), so that the behavior of ln*A*(*T*) can be approximated by (8):

8 $$lnA\left(T\right)=\lambda {E}_{A}\left(T\right),$$

where *λ* is the coefficient. We note that the general relaxation law (5) can also take the form (9)^14^:

9 $$\sigma ={\sigma }_{0}\left[1+{\left(f/{f}_{0}\right)}^{s}\right],$$

where *f*_0_ is the frequency of crossover from the power-law behavior to a virtually constant conductivity. Using (9), (5), and (7), it is easy to show that [ln*A* ∝ -*s*ln*f*_0_] and, hence, [ln*A* ∝ *E*_*A*_], so that the experimental observation (8) is justified. Thus, the results in (Fig. 4) and relationships (7) and (8) are self-consistent. Importantly, they demonstrate a close connection between conductivity relaxation and SP hopping. Although theoretical models for frequency dispersion of SP AC conductivity have not been developed thus far, the uncovered peculiar links (7), (8) evidence the absence of classical relaxation and reveal the influence of phonons and electron–phonon coupling on AC conductivity. The performed analysis of AC conductivity firmly corroborates SP transport.

Because intrinsic Jahn–Teller effects can naturally lead to SP localization in perovskite nickelates, charge transport by SP hopping is innate in these materials. At sufficiently high temperatures, delocalization may occur, and SP transport may vanish. However, it is likely that SPs exist not only in the low-temperature insulator phase but also in the high-temperature metal-like phase of nickelates, where SP hopping may principally contribute to deviations from the classical metal behavior of resistivity.

Importantly, SP mobility is determined by optical phonons, which, in turn, can be controlled by lattice strain. Therefore, the magnitude of DC conductivity, the shape of the temperature dependence of DC conductivity, as well as the magnitude and frequency relaxation of AC conductivity can exhibit high sensitivity to strain. Our findings explain many previous observations of strain-dependent magnitude of DC conductivity in epitaxial thin films. Furthermore, they promote precise control over the DC and AC conductivity using lattice strain, which can be produced not only by film-substrate epitaxial misfit and/or thermal mismatch, but also by chemical doping, external mechanical stress, or electric field. Additionally, because SP (de)localization and mobility depend on electric and/or magnetic field, the revealed SP transport may enable unconventional novel applications. We believe that for a comprehensive understanding of conductivity in nickelates as well as for fruitful employment of these materials, the formation, transport, and explicit role of self-trapped SPs should be further investigated.

## Conclusions

Small-polaron hopping transport was suggested for perovskite nickelates and experimentally proven in representative films of SmNiO_3_. AC electrical conductivity was investigated and analyzed as a function of temperature over the range *T* = 8–500 K and frequency over the range *f* = 1–10^6^ Hz in epitaxial strained SmNiO_3_ films. The studies revealed the hopping mechanism of transport, where the temperature dependence of the DC conductivity and the temperature dependence of the activation energy were demonstrated to be specific for the hopping of small polarons. The activation energy was also shown to depend on lattice strain, which confirmed the presence of electron–phonon coupling and small polarons. The frequency-dependent AC conductivity was verified to exhibit hopping-type relaxation, whose temperature-dependent parameters were found to be nonclassical, self-consistent, and directly connected to the small-polaron activation energy. The observations of the Holstein-type activation energy for hopping, nonclassical relaxation behavior, and nonclassical consistency between activation and relaxation corroborated small-polaron transport.

## Supplementary Information

Supplementary Figures.

## Data Availability

All data generated or analysed during this study are included in this published article and its Supplementary Information file.
