# Supplementary material for: Small-polaron transport in perovskite nickelates
Source: Sci Rep. 2023 Aug 1;13:12493. doi: 10.1038/s41598-023-39821-z (PMC10394062; doi:10.1038/s41598-023-39821-z)
Supplement: Supplementary file 1 — Supplementary Figures. [file 41598_2023_39821_MOESM1_ESM.pdf]

SUPPLEMENTARY INFORMATION

to

Small-polaron transport in perovskite nickelates

M. Tyunina,[1,2*] M. Savinov,[1] O. Pacherova,[1] A. Dejneka [1]

[1] *Institute of Physics of the Czech Academy of Sciences, Na Slovance 2, 18220 Prague, Czech Republic*

[2] *Microelectronics Research Unit, Faculty of Information Technology and Electrical Engineering, University of Oulu, P. O. Box 4500, FI-90014 Oulu, Finland*

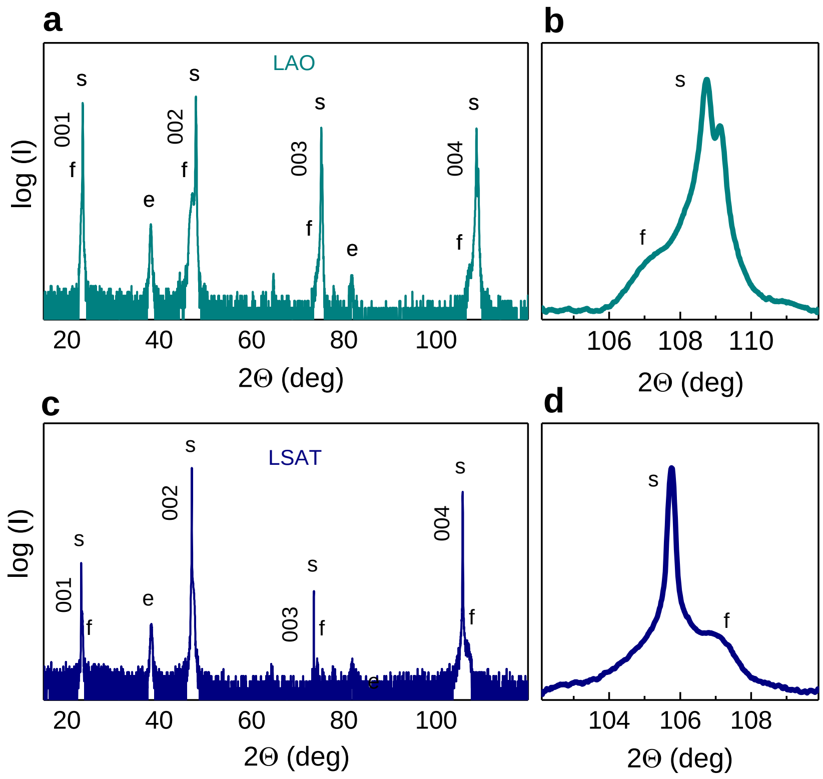

Fig. S1. XRD Θ-2Θ scans in the (a, b) SNO/LAO and (c, d) SNO/LSAT films. Peaks from substrates, films, and electrodes are marked by s, f, and e, correspondingly. In (b, d), details of the (004) peaks are shown.

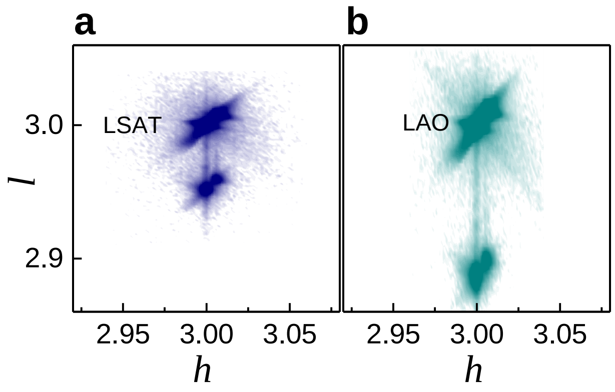

Fig. S2. Reciprocal space maps around (303) lattice points in the (a, b) SNO/LSAT and (c, d) SNO/LAO films. Coordinates are expressed in reciprocal lattice units of substrates.

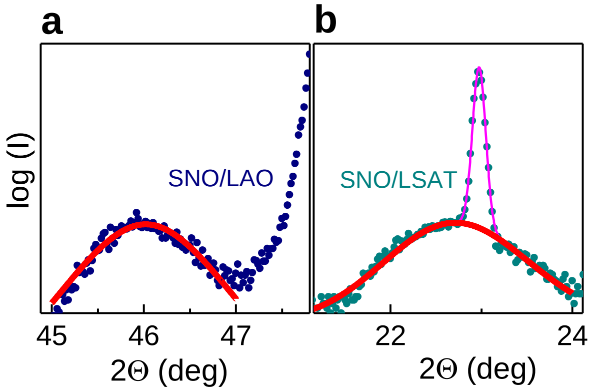

Fig. S3. XRD Θ-2Θ scans around the (a) (002) peak in SNO/LAO and (b) (001) peak in SNO/LSAT. Solid (red) curves show pseudo-Voigt fits.

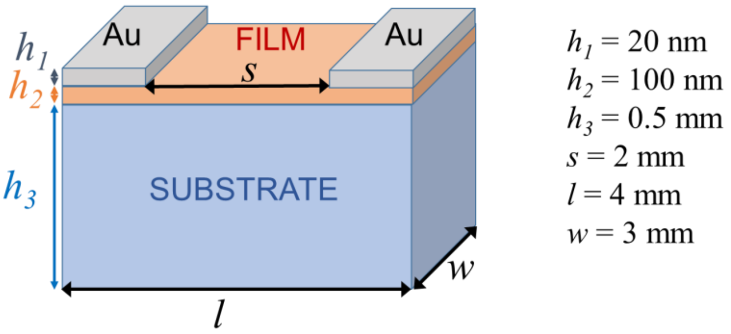

Fig. S4. Schematics of the sample for electrical characterization.

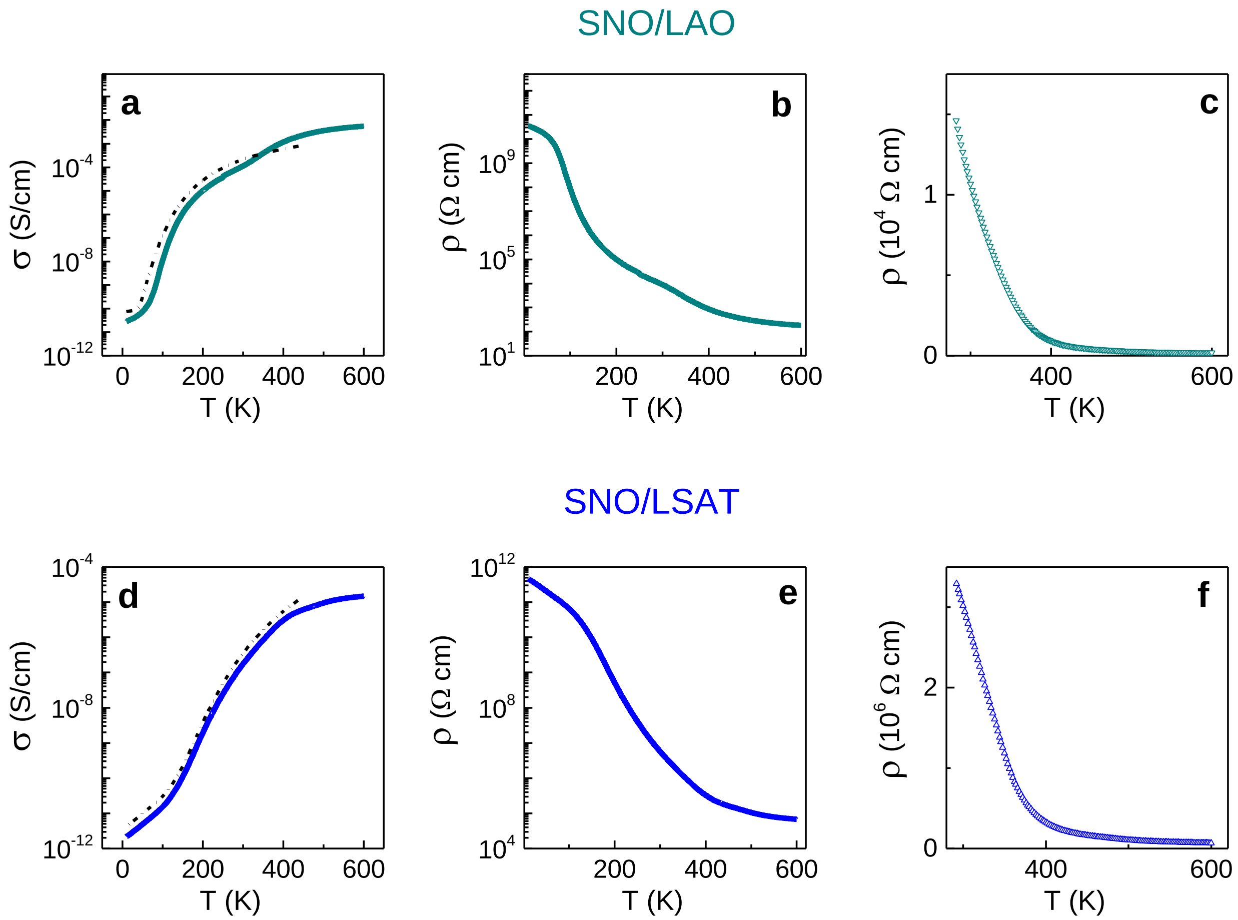

Fig. S5. DC conductivity (a, d) and resistivity (b-f) as a function of temperature in the (a-c) SNO/LAO and (d-f) SNO/LSAT films. Data are acquired by multimeter and smoothed using Savitzky-Golay method. In (a, d), dashed curves show AC conductivity at 10 Hz.

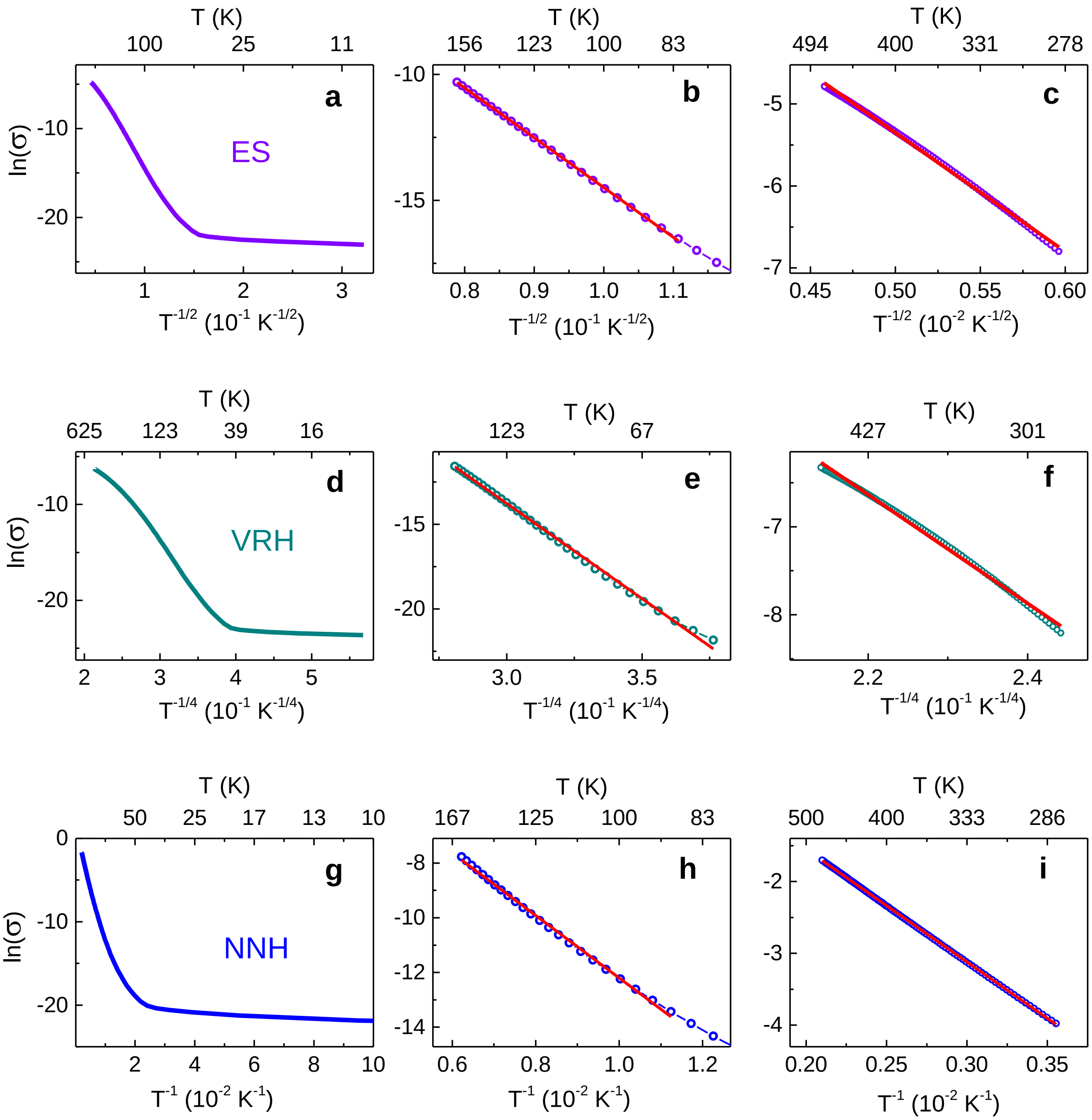

Fig. S6. Conductivity-temperature relationships for different hopping models in the SNO/LAO film. Straight lines show fits.

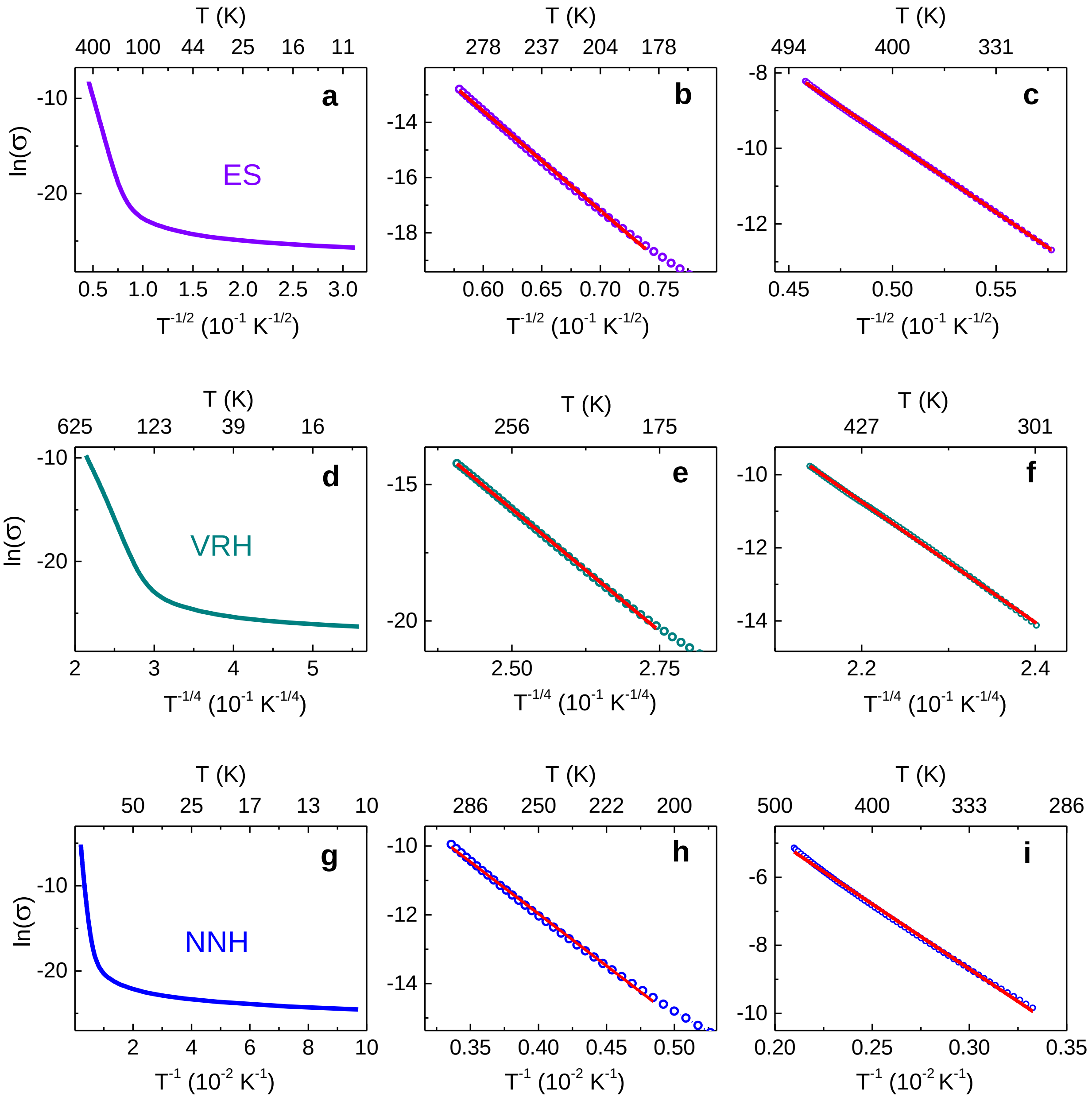

Fig. S7. Conductivity-temperature relationships for different hopping models in the SNO/LSAT film. Straight lines show fits.

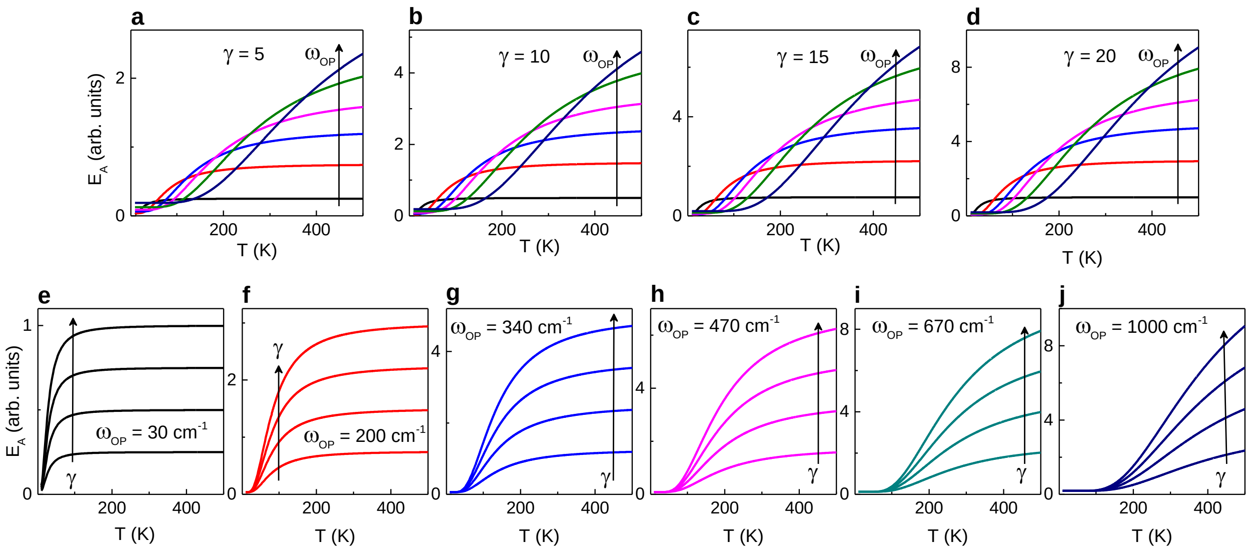

Fig. S8. Simulated activation energy for small-polaron hopping, E_A, as a function of temperature for different parameters ω_{OP} = 30-1000 cm^{-1} and γ = 5-20. Arrows show directions of parameter increase.

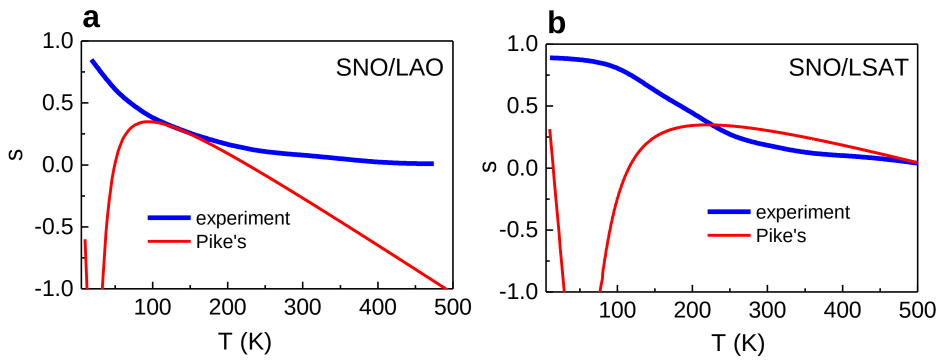

Fig. S9. Relaxation exponent *s* as a function of temperature in the (a) SNO/LAO and (b) SNO/LSAT films. Thick (blue) and thin (red) curves show experimental and calculated data, respectively.
